# Generative Deep Learning-Based Thermographic Inspection of Artwork

**DOI:** 10.3390/s23146362

**Published:** 2023-07-13

**Authors:** Yi Liu, Fumin Wang, Zhili Jiang, Stefano Sfarra, Kaixin Liu, Yuan Yao

**Affiliations:** 1 Institute of Process Equipment and Control Engineering, Zhejiang University of Technology, Hangzhou 310023, China; yliuzju@zjut.edu.cn (Y.L.); 2112102479@zjut.edu.cn (F.W.); jiangzl@zjut.edu.cn (Z.J.); 2 Department of Industrial and Information Engineering and Economics, University of L’Aquila, Piazzale E. Pontieri n. 1, Monteluco di Roio, I-67100 L’Aquila, Italy; stefano.sfarra@univaq.it; 3 Shanxi Key Laboratory of Signal Capturing & Processing, North University of China, Taiyuan 030051, China; 4 Department of Chemical Engineering, National Tsing Hua University, Hsinchu 300044, Taiwan

**Keywords:** artwork, infrared thermography, convolutional autoencoder, generative adversarial network, panel painting

## Abstract

Infrared thermography is a widely utilized nondestructive testing technique in the field of artwork inspection. However, raw thermograms often suffer from problems, such as limited quantity and high background noise, due to limitations inherent in the acquisition equipment and experimental environment. To overcome these challenges, there is a growing interest in developing thermographic data enhancement methods. In this study, a defect inspection method for artwork based on principal component analysis is proposed, incorporating two distinct deep learning approaches for thermographic data enhancement: spectral normalized generative adversarial network (SNGAN) and convolutional autoencoder (CAE). The SNGAN strategy focuses on augmenting the thermal images, while the CAE strategy emphasizes enhancing their quality. Subsequently, principal component thermography (PCT) is employed to analyze the processed data and improve the detectability of defects. Comparing the results to using PCT alone, the integration of the SNGAN strategy led to a 1.08% enhancement in the signal-to-noise ratio, while the utilization of the CAE strategy resulted in an 8.73% improvement.

## 1. Introduction

Artworks hold significant cultural and aesthetic value, and their conservation has gained increasing attention alongside improvements in people’s life quality [1]. However, artworks are prone to damage during production and preservation processes [2]. To address this issue, nondestructive testing (NDT) techniques are vital in early defect detection. NDT encompasses various methods, such as ultraviolet light, ultrasonic testing, X-ray imaging, and infrared thermography (IRT) [3,4,5,6]. Among these techniques, IRT has become a prominent method for the quality inspection of artworks because of its easy operation, rapid scanning capabilities, and ease of result interpretation [7].

In the IRT detection of artwork defects, the primary criterion relies on the temperature contrast between the area with defects and its surrounding regions in thermal images [8,9,10]. However, due to various factors, such as the equipment used for image acquisition and the experimental environment, the thermographic data obtained through IRT often contain noise, and defects may be obscured by the presence of an inhomogeneous background. Consequently, it becomes challenging to identify and discern defects solely through visual examination of these images. To address this challenge, there has been a growing interest in the development and application of thermogram processing and analysis methods to enhance defect detection in artwork.

In recent years, machine learning methods have shown remarkable performance in various data processing and analysis tasks, including those in the medical and industrial process domains [11,12]. For instance, Md et al. [12] utilized machine learning techniques to assess the quality of diverse production processes and provided an overview of the four industrial revolutions that revolutionized manufacturing. Machine learning algorithms have also found application in the domain of artwork defect detection, with principal component thermography (PCT) [13,14,15] being a commonly used method for enhancing defect detection and analyzing defect distributions. However, PCT itself has limitations in terms of its feature extraction capabilities, because the scarcity of thermal images further complicates the task of identifying optimal projection directions that effectively distinguish between defects and background elements. As a result, researchers have directed their attention towards exploring methods for enhancing thermographic data and investigating their performance in improving defect detectability. This area has emerged as a prominent research focus in the field, aiming to develop techniques that enhance the capabilities of PCT and enable more accurate and efficient identification of defects.

Deep learning, a prominent branch of machine learning, has garnered substantial attention and achieved remarkable success in the field of computer vision. Sampath et al. [16] proposed a deep learning-based model for gait-based fall prediction, which aids in the early identification of falls among individuals with walking disabilities. Generative deep learning methods, among the various approaches, have emerged as promising tools for image processing tasks [17,18,19,20,21]. These methods have shown potential in generating realistic and high-quality images, enhancing the field of computer vision and image analysis. By leveraging the power of generative deep learning, researchers have been able to tackle complex challenges and extract valuable insights from visual data. The autoencoder (AE) [22], a type of unsupervised generative model, has proven to be effective in various tasks, including image enhancement, noise reduction, nonlinear dimensionality reduction, and feature extraction. Another notable generative model in the field of unsupervised learning for image processing is the generative adversarial network (GAN) [23,24,25,26]. GANs have shown impressive results by employing a game-like learning approach between the generator and discriminator models. This mutual learning process enables GANs to generate high-quality output images. In the context of artwork defect detection, the application of generative deep learning strategies is expected to enhance both the quantity and quality of thermal images. This improvement can subsequently be combined with PCT to enhance the visibility of defects. However, it is worth noting that, to the best of our knowledge, this particular strategy has received limited investigation in the context of internal defect detection tasks in artwork.

In this study, we employed two different strategies, namely SNGAN and CAE, to process the data with the objective of comparing their respective effects on PCT results. SNGAN, a generative adversarial network, optimizes and refines the generated images through the iterative competition between its generator and discriminator models, effectively addressing the challenge of limited data availability. On the other hand, CAE enhances the images to mitigate the impact of the noise on defect visibility, resulting in improved PCT outcomes. By comparing the PCT analysis results obtained from the two image enhancement strategies with those derived from the original data, a comprehensive assessment can be conducted to evaluate the performance improvements achieved using these two techniques. The scientific contributions of this work are:

(1) A defect inspection method for artwork is proposed, which incorporates deep learning approaches for thermographic data enhancement. The integration of the proposed method and principal component thermography allows for improved detection of defects in artwork.
(2) The performance of two distinct generative deep learning approaches, namely SNGAN and CAE, is compared and analyzed. By examining their effectiveness in enhancing the detectability of artwork defects, we gain insights into the strengths and limitations of each approach.
(3) The proposed method is evaluated on a dataset comprising a panel painting. Experimental results demonstrate the validity of our approach in enhancing the quality and quantity of thermographic data, leading to improved defect detection. Furthermore, quantitative evaluation results provide evidence of the method’s efficacy in terms of enhancing the signal-to-noise ratio and F-score.

The remainder is organized as follows. Section 2 describes the data acquisition process. Section 3 presents the framework and implementation of the two deep learning methods for image enhancement. Section 4 discusses the dataset used in the experiments and provides a comparative analysis of the experimental results, along with a discussion of the underlying factors contributing to the observed differences. Finally, Section 5 concludes the work, summarizing the findings and outlining potential directions for future research.

## 2. Related Works

### 2.1. Thermographic Data and Preprocessing

**Figure 1:**
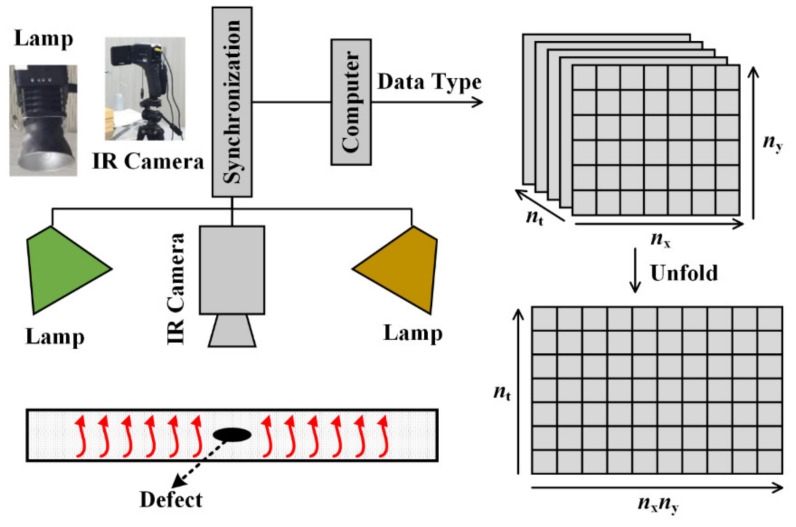
IRT detection device.

Data acquisition is a fundamental step in defect detection experiments, serving as the foundation for subsequent analysis. IRT detection systems are commonly employed to acquire thermal image data. In this study, the laboratory setup for data acquisition typically consists of the sample under investigation, two lamps, an infrared camera, and a computer for subsequent processing. The configuration of the IRT device is depicted in Figure 1. During data acquisition, the lamps are used to stimulate the sample, and the infrared camera captures the surface temperature distribution of the sample at each time point during the cooling phase. The temperature contrast at different locations reveals the characteristics of the inspected sample, influenced by the varying materials and the presence of defects.

The thermographic data acquired from the IRT detection system can be represented as a three-dimensional (3D) matrix, denoted as $n_{t}\times n_{x}\times n_{y}$. Here, $n_{t}$ corresponds to the number of thermal images, and $n_{x}\times n_{y}$ represents the total number of pixels per image. However, for further analysis, it is necessary to convert the 3D matrix into a two-dimensional (2D) matrix to facilitate processing. This transformation involves expanding the aforementioned 3D matrix into a 2D matrix, where each row represents a thermal image, and each column represents the temperature variation in a pixel.

### 2.2. Principal Component Thermography

Principal component analysis (PCA) is a widely used multivariate statistical analysis method that aims to reduce the dimensionality of data. Using linear transformations, PCA extracts the primary features of high-dimensional data and projects them onto a lower-dimensional space. This technique offers various advantages, including simple implementation, feature extraction, and noise reduction. In the field of IRT, PCT [13,14,15] employs a singular value decomposition of the covariance matrix of the thermograms to accomplish thermographic data dimensionality reduction and capture significant changes in the data. In the meantime, by projecting the original data onto the directions of maximum variance, PCT effectively reduces noise contained in the thermographic data.

The extraction of the first principal component (PC) can be expressed as follows:

(1) $$\begin{array}{l} \underset{p}{\max}{\left\|Xp\right\|}_{2}\\ \mathrm{subject}\mathrm{to}\;{\left\|p\right\|}_{2}\leq 1 \end{array}$$

Here, **X** represents the column-centered thermographic data matrix, **p** is a vector whose dimensions are $n_{x}n_{y}\times 1$, and ${\left\|\cdot \right\|}_{2}$ denotes the L2 parametrization. In PCT, the singular value decomposition method is commonly employed to solve each PC. The process begins by calculating the covariance matrix (**C**) of the thermographic data matrix (**X**). The eigenvalue decomposition of **C** is then performed, resulting in the following expression:

(2) $$\begin{array}{c} C=U\sum V^{T}\\ \sum =diag(\sigma_{1},\sigma_{2},\sigma_{3},\ldots ,\sigma_{p}) \end{array}$$

where **U** is an orthogonal matrix of order *m*, **V** is an orthogonal matrix of order *n*, **Σ** is an *m* × *n* rectangular diagonal matrix consisting of non-negative diagonal elements arranged in descending order, and $\sigma_{i}$ represents the singular value of matrix **C**. The singular values are sorted from largest to smallest, and the top-*k* singular values are selected. The corresponding *k* eigenvectors {**p***_i_*, *i* = 1, …, *k*} are obtained. Therefore, $t=Xp_{1}$ signifies the first PC, which is a linear combination of the columns in **X** and aims to explain the maximum possible variation in the data. Subsequent PCs can be calculated successively. In practice, only a few PCs are typically needed to capture the majority of the variance present in the data.

## 3. Methodology

In the context of defect analysis in thermographic data, relying solely on PCT may not yield satisfactory results. To achieve improved performance, a promising approach is to integrate deep learning methods with PCT. By leveraging deep learning techniques to expand and enhance the data, the performance of PCT in detecting defects is enhanced. In this study, we propose a defect inspection method for artwork that combines PCT with two different deep learning strategies for thermographic data enhancement. The framework and implementation steps of the proposed approach are outlined.

### 3.1. Problem Description

In order to detect defects in artwork using IRT, specifically pulsed thermography, the test sample is subjected to instantaneous heating using flash lamps. A thermal imaging camera is then used to capture temperature information on the surface of the sample during the subsequent temperature drop. Finally, a computer is utilized for data storage and analysis. The temperature signal at a specific pixel location in the thermal images can be described by the one-dimensional Fourier diffusion equation:

(3) $$T(s,t)=T_{0}+\frac{Q}{e\sqrt{\pi t}}\exp(-\frac{s^{2}}{4\alpha t})$$

where *T*(*s*,*t*) denotes the temperature at time *t* and depth *s*, *T*_0_ is the initial temperature, *Q* denotes the energy absorbed by the material surface from the heat source, and *α* and *e* represent the thermal diffusivity and thermal storage coefficient, respectively.

It is important to note that the aforementioned equation is derived under the assumption that the tested sample is a semi-infinite solid and that thermal diffusion is strictly one-dimensional. However, in reality, thermal diffusion in materials is often complex and three-dimensional, leading to nonlinearities in the obtained thermal images. In addition, issues, such as experimental noise and scarcity limiting the availability of thermal images, make it challenging for PCT to identify an effective projection direction, impeding its ability to achieve accurate defect detection. Therefore, the objective of this study is to investigate how generative deep learning techniques can enhance the quality of thermographic data and improve the performance of PCT in defect analysis.

### 3.2. SNGAN-Based PCT Approach

Data augmentation is a crucial technique for addressing the challenge of limited data availability and reducing data collection costs. In thermal image data analysis, data augmentation methods play a vital role in improving the performance of thermal imaging techniques by increasing the diversity of thermal image data. GANs have emerged as a prominent approach for learning and generating data distributions [27] and have been successfully applied in various domains, including image synthesis, video generation, speech synthesis, and natural language processing [20,21,22,23], making them a focal point in the field of deep learning.

In this study, an improved GAN algorithm, spectral normalized GAN (SNGAN), is used to enhance thermographic data. Traditional GANs often face challenges, such as gradient disappearance or explosion during training, due to the complexity of the generator (*G*) and discriminator (*D*). SNGAN addresses these issues by employing spectral normalization, which normalizes the weight matrix in the *D* [28]. This normalization technique restricts the spectral radius of the weight matrix’s singular value decomposition to a fixed range, effectively preventing gradient disappearance or explosion. Furthermore, SNGAN offers simplicity and avoids the need for complex hyperparameter tuning. This regularization method enhances the training stability of GANs and helps generate higher-quality images.

**Figure 2:**
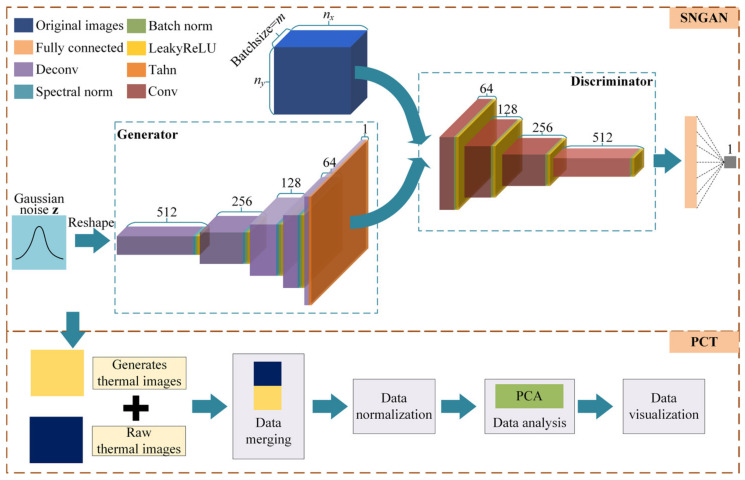
Framework of the GPCT model.

To ensure that SNGAN has sufficient discriminating and generating capabilities, we designed a *G* comprising one fully connected layer and five deconvolution layers and a *D* comprising one fully connected layer and four convolutional layers. The design of these layers is based on the size of the thermal images. The architecture of the SNGAN-based generative principal component thermography (GPCT) model is depicted in Figure 2.

In the network, the first deconvolutional layer employs a 3 × 3 convolutional kernel with a stride of 1, enabling the model to capture local information in the input features more effectively. The subsequent convolutional and deconvolutional layers employ 4 × 4 convolutional kernels with a stride of 2, enabling the model to capture global contextual information in the input features. LeakyReLU activation functions are utilized in all layers except for the last deconvolutional layer, where the activation function is Tanh. Notably, spectral normalization is applied to the weight matrix of each convolutional layer in *D*. In doing this, the model becomes more robust against input perturbations, enhancing the overall model robustness [29].

In the GPCT initial, $n_{g}$ new thermal images are generated using SNGAN and transformed into 2D data. These new images are then merged with the original dataset. This integration results in a new 2D dataset with a specific size $\left(n_{t}+n_{g}\right)\times n_{x}n_{y}$. Finally, this new dataset is fed into the PCT for subsequent processing and analysis.

### 3.3. CAE-Enhanced PCT Approach

Data enhancement methods can significantly enhance the performance of thermal imaging techniques by mitigating the impact of background noise on the data. CAE, one of the variants of AE, can compress input data into a low-dimensional representation and then reconstruct the input data from this representation, effectively learning meaningful data features. Normally, it is used for tasks, such as dimensionality reduction, denoising, and feature extraction. In particular, CAE has proven to be very effective for image enhancement tasks.

The encoding and decoding processes of CAE can be expressed as follows:

(4) $$Y=P(W_{1}*X+a_{1})$$

(5) $$Z=Q(W_{2}*Y+a_{2})$$

In the above equations, **X** denotes the thermographic data, **Y** represents the compressed data produced by the encoder, **Z** signifies the decoded data, $W_{1}$, $W_{2}$, $a_{1}$, and $a_{2}$ are the weights and biases of the encoder and decoder, respectively, P(⋅) and Q(⋅) are the nonlinear activation functions of the encoder and decoder, respectively, and ∗ denotes the two-dimensional convolution operation.

In this study, we developed the CAE-enhanced principal component thermography (CPCT) method for detecting internal defects in artworks. The CAE architecture consists of an encoder and a decoder. The encoder compresses the input data using a series of convolution and pooling operations, resulting in a lower-dimensional coded representation that captures the essential features of the input data. Subsequently, the decoder reconstructs the coded representation back into the original input data using convolution and up-sampling operations. The aim of the reconstruction process is to closely resemble the original input, thus generating reconstructed data that closely resemble the original data. Specifically, we set the size of the convolution kernel to 3, and the convolution stride was set to 1. The pooling operation utilizes maximum pooling with a kernel size and stride of 2. The encoder employs the ReLU activation function, while the output layer uses the sigmoid function, and the remaining layers utilize ReLU. The newly decoded data are then fed into the PCT model for dimensionality reduction, resulting in the extraction of *k* principal components. This process effectively highlights defects in the images.

**Figure 3:**
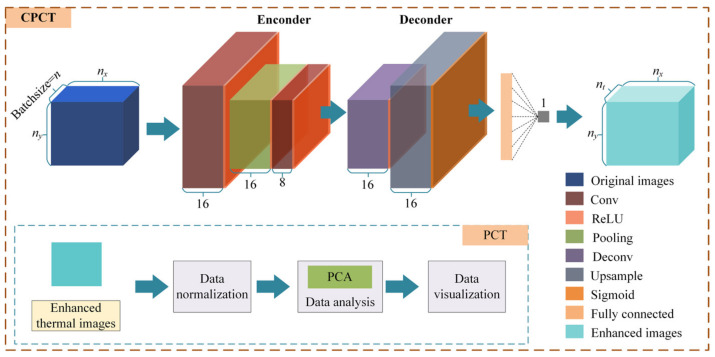
Framework of the CPCT model.

Figure 3 illustrates the framework of the CPCT model utilized in this study. The detection process based on CPCT is depicted in Algorithm 1.
**Algorithm 1:** Set n_components = *k*Input: 3D data $n_{t}\times n_{x}\times n_{y}$
Output: 2D data $k\times n_{x}n_{y}$
Algorithm Flow:  Step 1: Initialize model weights and biases;  Step 2: Encode the input data using the encoder and obtain the reconstructed output using the decoder;  Step 3: Measure the performance of the model in terms of the error between the reconstructed output and the original input, with a loss function of mean squared error;  Step 4: Calculate the gradient of the error with respect to the model parameters;  Step 5: Transfer of errors and calculation of gradients using a back-propagation algorithm;  Step 6: Minimize the reconstruction error of the model by updating the model parameters based on the gradient information using an optimization algorithm, Adam;  Step 7: Repeat steps 2 to 6 until the training is completed or the preset number of iterations is reached;  Step 8: Transform the trained 3D data into a 2D matrix **A**;  Step 9: Perform column normalization on the 2D matrix **A**;  Step 10: Perform PCT on the normalized 2D matrix **A** to obtain 2D data $k\times n_{x}n_{y}$.

After applying Algorithm 1, each row of the obtained data matrix is transformed into a 2D matrix with dimensions $n_{x}\times n_{y}$ and can be visualized. By observing the obtained images, it is convenient to identify defect information within the sample. Defects in the artwork can be detected by analyzing the patterns and irregularities present in these visual representations.

## 4. Experiment

To objectively evaluate the assay’s performance, signal-to-noise ratio (SNR) [30] is a widely used metric in thermography data processing. In this study, SNR is employed to assess the effectiveness of the proposed approach. It is calculated as follows:

(6) $$\mathrm{SNR}=\frac{\left|M_{\mathrm{def}}-M_{\mathrm{in}}\right|}{\sigma_{\mathrm{in}}}$$

In the above equation, $M_{\mathrm{def}}$ denotes the average pixel value of the defective region, $M_{\mathrm{in}}$ represents the average pixel value of the intact region, and $\sigma_{\mathrm{in}}$ is the standard deviation of the pixel values in the intact region. SNR measures the thermal contrast between the defective and non-defective regions. A larger SNR value indicates a higher level of defect information in the image, indicating better performance of the assay.

The F-score is a commonly used evaluation metric in classification, diagnosis, and image processing tasks [31,32]. In the context of defect analysis, it is utilized to assess the performance of the model by considering four scenarios: true positive (TP), false positive (FP), true negative (TN), and false negative (FN). Specifically, the F-score is calculated as follows:

(7) $$F=\frac{\left(\beta^{2}+1\right)\times P\times R}{\beta^{2}\times P+R}$$

where β is a default value for the associated precision and recall weights, *P* = TP/(TP + FP) represents detection precision, and *R* = TP/(TP + FN) represents detection recall. A higher F-score indicates better defect visibility, implying superior performance of the corresponding thermography method.

### 4.1. Preparation and Pretreatment of Samples

**Figure 4:**
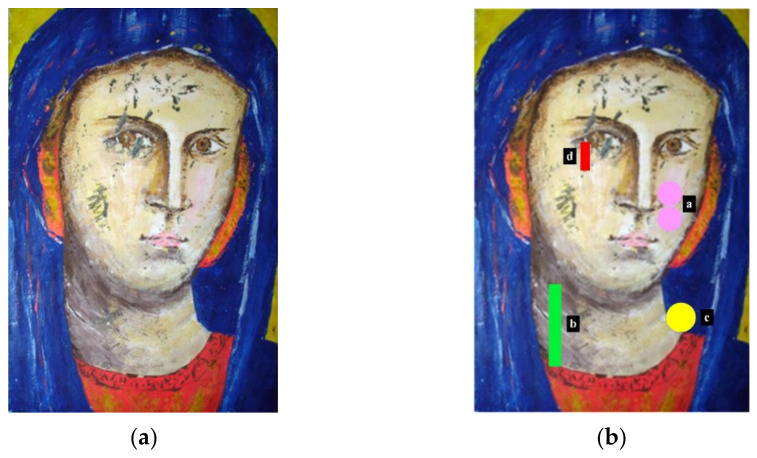
Madonna sample and defect distribution: (**a**) Madonna sample; (**b**) defect distribution diagram.

In this study, an experimental test was conducted using a panel painting sample named “Madonna”, as depicted in Figure 4a. The objective was to compare the performance of different data enhancement methods. The Madonna sample consists of four defects; however, the collected data could only capture three of these defects. Therefore, only these three defects were utilized in this study for calculating the SNR of each method.

The sample has dimensions of 15 × 21 × 2 cm and is composed of poplar wood with a layered structure consisting of a bottom layer of canvas, plaster, and glue. A varnish layer was added to protect the painting layer. Artificial zones, simulating defects at different depths, were incorporated within this structure. The defects in the Madonna sample, as shown in Figure 4b, were simulated by inserting Mylar^®^, also known as BoPET (biaxially oriented polyethylene terephthalate), sheets. In Figure 4b, the a, b, c and d represent different defects. The shape, size, and depth of each defect are outlined in Table 1. The heating device involves two 250 W lamps (Siccatherm E27, OSRAM) positioned 50 cm apart from each other and 48 cm from the painting. An infrared camera with a frame rate of 1 frame per second was employed to capture a total of 270 thermal images. Among these, the first 90 images correspond to the heating phase, while the remaining 180 images represent the cooling phase. The region of interest (ROI) size was set to 240 × 320 pixels for each thermal image. Consequently, an infrared thermal imaging dataset with dimensions of 270 × 240 × 320 was obtained. To facilitate subsequent analysis, the dataset was transformed into a 2D matrix with dimensions of 270 × 76,800. Each row of the matrix corresponds to a thermal image, while each column represents the temporal change in pixel values.

**Figure 5:**
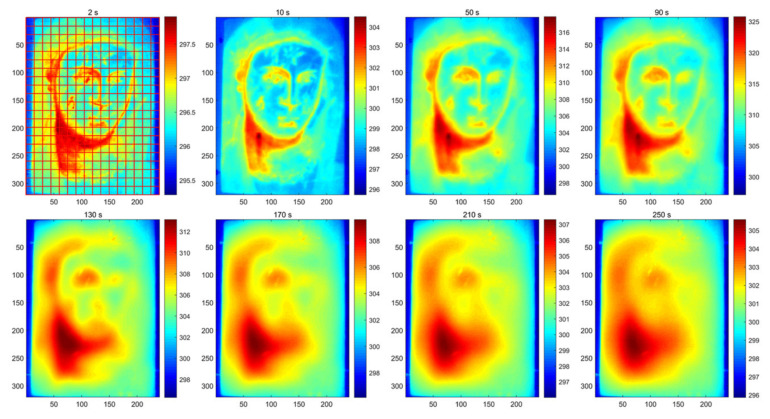
Original thermal images, where the grid division for F-score calculation is illustrated in the first sub-figure.

**Figure 6:**
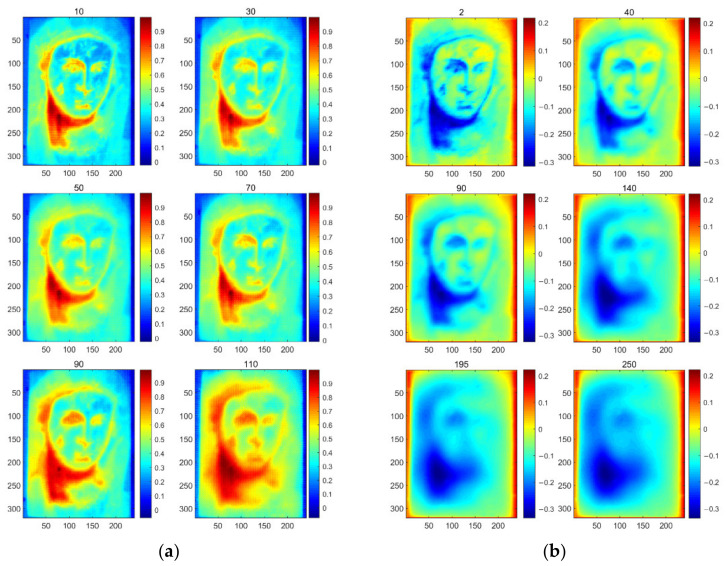
Results of image enhancement based on deep learning strategies: (**a**) generated images; (**b**) enhanced images.

Figure 5 displays the thermal images obtained using the IRT system at eight different time instances, providing an overview of the image quality during the early, middle, and late stages of data acquisition. In these images, the presence of defects is not readily apparent. Therefore, additional methods must be applied to uncover the hidden defects beneath the oil painting. To address this, the original thermal images are processed using two different strategies of image enhancement. Figure 6a depicts the generated images of SNGAN, while Figure 6b shows the enhanced images of CAE. Some of the parameters are set by default, such as the optimizer and the multiplicative factor for spectral normalization in SNGAN. Other parameters were determined through several trials, and their specific values were set as follows:

For the training process of SNGAN:
Epoch: 500
Batch size: 30
Learning rate: 0.0002
Dropout: 0.25
For the training process of CAE:
Epoch: 50
Batch size: 30
Learning rate: 0.0002

### 4.2. Results and Discussion

**Figure 7:**
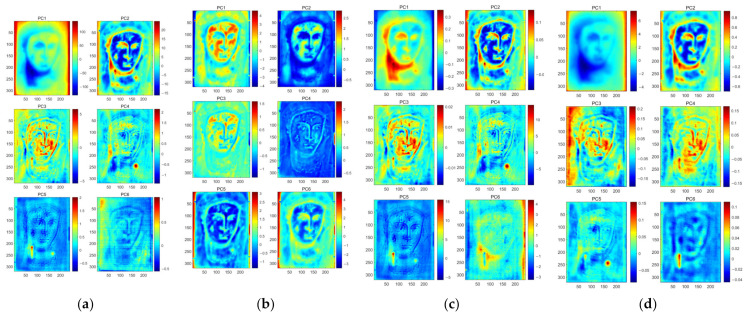
Comparison of different methods: (**a**) PCT; (**b**) SPCT; (**c**) GPCT; (**d**) CPCT.

Next, PCT was applied to analyze the original thermal images, the enriched thermographic dataset obtained using SNGAN, and the enhanced thermal images generated through noise reduction with CAE. The analysis results are represented as PCT, sparse PCT (SPCT), GPCT, and CPCT, respectively, in Figure 7. In the case of GPCT, the rich dataset consists of original images and generated images, resulting in 390 thermal images in the total dataset.

Upon observing and analyzing the three different outcomes, it is evident that the defects, particularly defect a and defect b, are more prominently highlighted in GPCT compared to the background. However, CPCT effectively reduces the influence of the background and enhances the visibility of defect information. These results can be attributed to the complex background information in the Madonna dataset, which significantly affects the detection of defects. During the augmentation process, the model tends to focus more on the background information rather than learning the characteristics of defects, leading to suboptimal results after augmentation. In contrast, the dataset enhanced using the CAE model exhibits reduced noise, making it easier to distinguish defects from the background. The CAE-based enhancement approach effectively preserves and enhances the defect information while minimizing the impact of noise and background interference. These findings align with the qualitative results, demonstrating the advantages of CAE-based enhancement in improving defect detection. The SNR values provided in Table 2 further support the qualitative results. The SNR values of CPCT were higher than those of the other three methods for both individual defects and all defects. A reasonable explanation is that the CPCT model uses the CAE data enhancement strategy to reduce the noise in the original image and obtain the essential characteristics of the defects. For instance, in the case of defect b, PC6 of CPCT exhibits a smoother background, which effectively enhances the differentiation between the background and the defect. These findings indicate that CPCT not only outperformed the other methods in terms of SNR values but also yielded improved visual results in distinguishing defects from the background. These quantitative and qualitative analyses further support the efficacy of CPCT in enhancing defect detection performance.

Table 3 presents the results of the relative improvement rates achieved by combining SNGAN or CAE with PCT. The relative improvement rates for defects a and b are relatively low when SNGAN is combined with PCT alone, and defect c shows a negative effect. This could be attributed to SNGAN’s tendency to primarily learn the raw thermal image data, including the heavily inhomogeneous background, which may result in suboptimal performance in defect analysis when combined with traditional PCT. In contrast, the CPCT model demonstrates high SNR defect enhancement rates for each defect detection, with the highest rate reaching 121.98%. This can be attributed to the CPCT model’s utilization of the CAE data enhancement strategy, which reduces noise in the original image and captures the essential features of the characterized defects. These findings support the effectiveness of the CPCT model in enhancing defect visibility through the combination of CAE-based data enhancement and PCT analysis.

**Figure 8:**
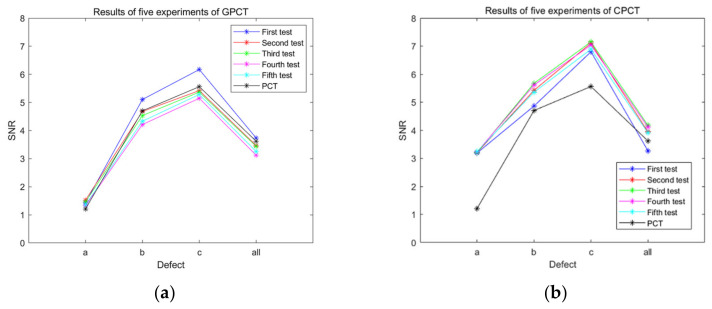
Comparison of the SNR values of the five rounds of test results of the proposed model with the PCT results: (**a**) GPCT; (**b**) CPCT.

In order to validate the results obtained from the proposed artwork defect detection model, we calculated the SNR values for the five rounds of test results and compared them with the results obtained from PCT on raw thermograms. Figure 8, presented below, illustrates the results of the five tests conducted for each model. Despite the inherent uncertainty in the training process, the consistency of the results across the five tests indicates the validity and reliability of the proposed models. This comparison serves to demonstrate that the proposed artwork defect detection model yields consistent and accurate results, providing confidence in its effectiveness for detecting defects.

In Figure 5, the first sub-figure illustrates how the images are divided into cells for calculating *P* and *R* values. Subsequently, using Equation (7), Table 4 showcases the F-score values obtained through different methods. The proposed CPCT model achieved the highest F-score values for all three types of defects, demonstrating its strong defect identification capability. This improvement is attributed to the utilization of data enhancement strategies in the CPCT model, which enhances the quality of the data used for modeling. In the context of this work, existing approaches primarily concentrate on the analysis and processing of thermographic data using machine learning methods to attain specific objectives, such as noise removal, mitigation of inhomogeneous backgrounds, and extraction of defect features. However, it is worth noting that future advancements may involve the incorporation of physical knowledge-based models. Specifically, physics-informed neural networks are a promising technique, which leverage both machine learning techniques and the underlying physics principles associated with heat transfer.

## 5. Conclusions

In this study, we employed two deep learning strategies, SNGAN and CAE, for enhancing thermographic data in order to improve the accuracy of defect detection in panel paintings. The SNGAN strategy facilitated the generation of additional thermal images, while the CAE strategy focused on denoising and deblurring. The PCT results based on the enhanced thermographic data are significantly better than those based on the raw thermograms. Specifically, CPCT outperforms GPCT in the case study discussed in this paper. The results of our study indicate that deep learning-based image enhancement techniques hold great potential for advancing defect detection and evaluation in the conservation of artworks. When comparing the results of PCT alone with the proposed CPCT method, it is observed that the CPCT method achieved a significant improvement in the signal-to-noise ratio (SNR) for identifying all defects in the artwork. Specifically, the CPCT method increased the SNR value by 8.73% compared to PCT alone. Furthermore, when evaluating individual defects, the CPCT method showed a substantial enhancement in performance. The highest F-score value achieved using the CPCT method improved by 49.46% compared to PCT. Furthermore, this proposed framework could also be applicable for the inspection of augmented-reality-generated content [17,18]. Looking ahead, further research will be conducted to explore the combination of deep learning strategies for thermal image enhancement with other feature extraction methods. This will contribute to the development of more comprehensive and effective approaches for defect detection and analysis in various domains.

## Figures and Tables

**Table 1:** Defect information of Madonna sample.

Number	Shape	Area(mm^2^)	Depth(mm)
a	Two Circles	*d* = 5	0.3–0.8
b	Rectangle	28 × 4	0.5–1.1
c	Circular	*d* = 8	0.7–1.6
d	Rectangle	14 × 3	2.0

**Table 2:** SNR values of different methods.

	SNR			
	Defect a	Defect b	Defect c	All Defects
PCT	1.210	4.703	5.561	3.619
SPCT	1.745	1.051	1.273	1.063
GPCT	1.535	4.887	5.438	3.658
CPCT	**2.686**	**5.264**	**6.544**	**3.935**

**Table 3:** Comparison of the relative improvement rates of SNR using different methods.

	Improvement Rate Compared to PCT			
	Defect a	Defect b	Defect c	All Defects
GPCT	26.86%	3.91%	−2.21%	1.08%
CPCT	**121.98%**	**11.93%**	**17.68%**	**8.73%**

**Table 4:** F-score of different methods.

	F-Score		
	Defect a	Defect b	Defect c
PCT	0.632	0.640	0.923
SPCT	0.769	0.640	0.889
GPCT	0.632	0.640	0.727
CPCT	**0.857**	**0.957**	**1.000**

## Data Availability

Not applicable.
